# Comparing the hippocampal volumetric atrophy between demented and nondemented individuals with Parkinson's disease: A systematic review and meta‐analysis

**DOI:** 10.1002/hsr2.1514

**Published:** 2023-09-11

**Authors:** Mohammad Yazdan Panah, Yousef Mokary, Sangam Shah, Sangharsha Thapa, Swati Chand, Vahid Shaygannejad, Omid Mirmosayyeb

**Affiliations:** ^1^ Isfahan Neurosciences Research Center Isfahan University of Medical Sciences Isfahan Iran; ^2^ Institute of Medicine Tribhuvan University Maharajgunj Nepal; ^3^ Kathmandu University School of Medical Sciences Dhulikhel Nepal; ^4^ Rochester General Hospital Rochester New York USA

**Keywords:** dementia, hippocampal atrophy, Parkinson's disease, volumetry

## Abstract

**Background:**

Parkinson's disease (PD) is one of the most common neurodegenerative conditions in the world and, when combined with dementia, can lead to immense cerebral volume loss. Of significant importance among all cerebral regions, is the hippocampus. This region plays a pivotal role in memory, and understanding its pathological alterations can answer vital questions regarding dementia. As such, we designed this study to compare the hippocampal volumes of PD patients with dementia (PDD) versus PD without dementia.

**Methods:**

PubMed, Web of Science, Scopus, and Embase were searched for relevant studies. We also searched the references sections of all included studies. The original search began in March 2022 and was extended to the end of July 2022. All related data were extracted from the studies. If the studies were conducted on patients from comparable patient groups, the most recent study with the most extensive data set would be included.

**Results:**

A statistically significant difference was observed comparing the raw hippocampal volumes in participants with PDD and PD (*p* value = 0.01). In a comparison of normalized hippocampal volume between PDD and PD, there was a statistically significant difference (*p* value < 0.01), as well.

**Conclusion:**

Although further research is required to illuminate the temporal relation between the onset of dementia and hippocampal atrophy in demented PD individuals, the present study highlights the importance of utilizing volumetric studies on memory‐related cerebral regions to diagnose the initiation of dementia in the early stages.

## Key points

The present study highlights the importance of utilizing volumetric studies on memory‐related cerebral regions to diagnose the initiation of dementia in the early stages. Compare the hippocampal volumes between Parkinson's disease patients with and without dementia.

## 1 INTRODUCTION

Parkinson's disease (PD) is a chronic degenerative disease of the central and peripheral nervous system that can affect a variety of people worldwide. PD patients suffer from a wide range of motor dysfunctions, including resting tremors, rigidity, bradykinesia, and postural instability. PD is the second most prevalent neurodegenerative disease after Alzheimer's disease and is characterized by the loss of dopaminergic neurons in the substantia nigra and the presence of alpha‐synuclein‐containing inclusion bodies in the surviving neurons. The prevalence of PD in the general population is 0.3%, and 1%–3% in the elderly population.^1^, ^2^, ^3^

Cognitive impairment has recently been recognized as an important clinical manifestation of PD. Approximately 30% of individuals with PD develop PD dementia (PDD). PDD is largely characterized by executive dysfunction, a consequence of dopaminergic dysfunction in the frontostriatal circuit.^4^, ^5^ A study estimated that 3.6% of dementia cases in the population are related to PD. PD and dementia have a strong relationship, as demonstrated by a study that followed patients for over 20 years and found cumulative incidence rates of over 80%.^2^ It is well known that the age at onset, the patient's age, the duration, and the severity of the disease are some risk factors that accentuate the risk of dementia in patients with PD. The presence of rapid eye movements and autonomic dysfunction contributes significantly to the risk of PDD.

Degeneration of the amygdala and hippocampal structures has been observed in PD patients suffering from dementia. As a result, cognitive impairment and Lewy neurite density in the CA2 hippocampal field are associated with cognitive impairment, leading to a possible link between dementia in PD and disruption of connections between the entorhinal cortex, septal nuclei, hypothalamus, and CA1 fields. It is becoming increasingly clear that the loss of hippocampal volume in PD is associated with cognitive impairment and dementia because the loss of hippocampal volume was recently identified as a contributor.^6^, ^7^

Recent improvements in magnetic resonance imaging (MRI) hardware and acquisition methods provide considerable potential for more sensitively examining alterations in the brain with aging. Structural imaging has historically been used to “rule out“ intracerebral lesions as the cause of dementia, but nowadays, it is utilized to confirm the diagnosis.^8^ With the recent introduction of new dementia treatments and the importance of subtype‐specific management, there is currently a resurgence of interest in the use of brain imaging techniques that could aid in the accurate diagnosis of PDD, Alzheimer's disease, vascular dementia, mixed dementia (MD), and normal pressure hydrocephalus.^6^, ^9^ MRI is the main method for assessing the relationship between hippocampal volume and PDD.

Several studies have measured the extent of hippocampus atrophy in aging and dementia, although the results are inconsistent. Studies on dementia and hippocampus volume decrease in older people with intact cognitive function are few and far between. In this study, we measure the amount of hippocampus volume loss associated with aging and dementia and investigate how dementia affects brain atrophy.

## 2 METHODS

### 2.1 Literature search

**Figure 1:**
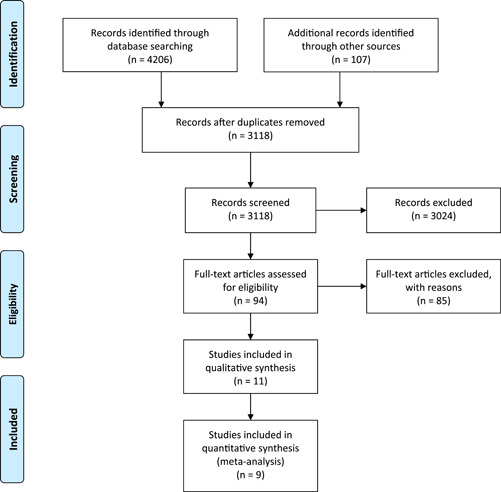
Flow diagram summarizing the criteria for selecting eligible studies under PRISMA. PRISMA, Preferred Reporting Items for Systematic Reviews and Meta‐Analyses.

Until July 2022, the following electronic databases were searched for pertinent studies: PubMed, Web of Science, Scopus, and Embase. Volume AND ([Lewy Body Parkinson's Disease] OR [Primary Parkinsonism] OR [Parkinson's Disease] OR [Idiopathic Parkinson Disease] OR [PD] OR [Parkinsonism]) AND [dementia] were the search terms employed. We excluded the other causes of Parkinsonism from our results due to the fact that PD is the most common cause of parkinsonism, but not the only cause. We also searched the references sections of all included studies. The original search began in March 2022 and was extended to the end of July 2022. This study was conducted according to the Preferred Reporting Items for Systematic Reviews and Meta‐Analyses (PRISMA) guidelines (Figure 1).

### 2.2 Criteria for inclusion/exclusion

All studies with the following information were included: (1) they were published in English as a peer‐reviewed article; (2) they investigated the MRI data among PD patients with dementia and PD patients without dementia; (3) they used volumetric analysis of structural MRI data; (4) or could have been extracted or retrieved these data from the authors. In our study, those in the cohort and longitudinal studies incorporating this review at one time were included, as well as cross‐sectional studies.

Studies were excluded if (1) there was not enough information to determine how many people were in each group, (2) Neither the PD patients with dementia group nor the PD patients without dementia group contained five or more participants, and (3) the comparison groups consisted of individuals with neurological disorders, (4) letter, abstract, protocol, review, systematic review, meta‐analysis, or case report studies and books.

### 2.3 Data extraction

Authors, year of publication, demographic factors (number of subjects, age at baseline, and gender), illness factors (Hoehn and Yahr Scale Score, studied region), and mean and standard deviation of hippocampal volume were among the information taken from the studies. If the studies were carried out by the same research team on patients from comparable patient groups, the most recent study with the most extensive data set would be included.

### 2.4 Risk of bias

Using a modified ROBINS‐I (Risk of bias in non‐randomized studies‐I) methodology in the following domains, authors A.B. and S.T. evaluated the risk of bias among the included papers. (2) Confounding bias (3) exposure verification (4) outcome measurement (5) missing data (6) result reporting. A study's bias risk was rated as low if all of its domains had low risk, moderate if any one of them had moderate risk, serious if at least one of them had serious risk, and critical if one of them had critical risk. Studies were neither added nor removed based on a risk of bias analysis.

### 2.5 Statistical analysis

One author used STATA version 17^10^ to do a meta‐analysis utilizing the data that had been gathered. Using *I* ^2^ statistics, the heterogeneity between the studies was explored. We classified low, moderate, and high heterogeneity as *I* ^2^ values of 25%, 25%–50%, and >50%, respectively. Similarly, a random‐effects model was chosen based on the *I* ^2^ statistic's indication of heterogeneity. The heterogeneity among the selected studies was confirmed using the Chi‐square test. Utilizing the Der Simonian and Laird approach, “tau^2^” was calculated.

The effect sizes on the distinction between volume in PD patients with dementia and PD patients without dementia were expressed using the standardized mean difference (SMD). To compare PD and PDD in terms of hippocampal volume, we used either the total or the mean hippocampal volume. According to the study population's geographic distribution and the detection method, subgroup analyses could not be carried out. The creation of forest patches was the last step in the interpretive process. A statistically significant finding was one with *p* 0.05. The visual inspection of funnel plots with SMD on the horizontal axis and standard error on the vertical axis was used to detect publication bias. Similar to the previous example, sensitivity analysis was done by deleting one study at a time to examine the impact of larger studies on the pooled effect.

## 3 RESULT

### 3.1 Selection of studies

As a result of the description of selecting and omitting articles, a PRISMA flow chart was developed (Figure 1). First, 4206 studies were retrieved using this search literature. After eliminating duplicates, 3118 remained. As a result of the screening, 94 studies were identified that could potentially be eligible, and in the meta‐analysis, 7 original studies met the inclusion criteria. Hand‐searching and checking the references yielded two additional studies.

### 3.2 Study characteristics and significant findings of the included studies

**Figure 2:**
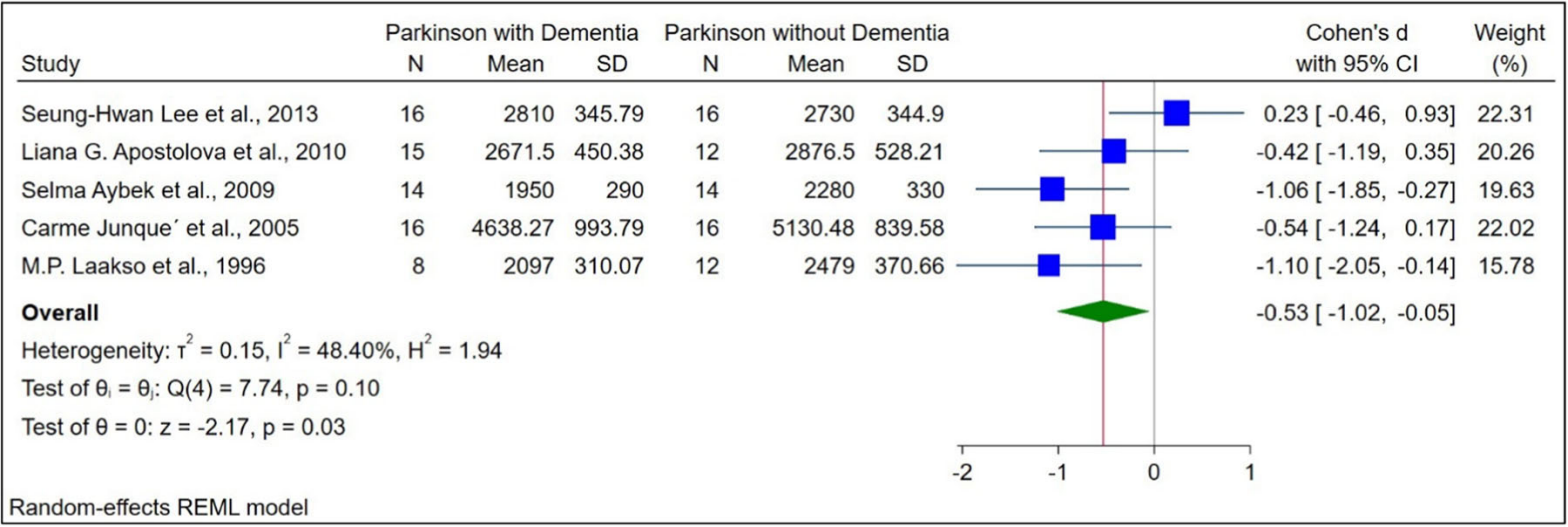
The forest plot of comparison of raw hippocampal volume between PDD and PD, illustrating statistical differences in the hippocampal volume. PD, Parkinson's disease; PDD, Parkinson's disease with dementia.

**Figure 3:**
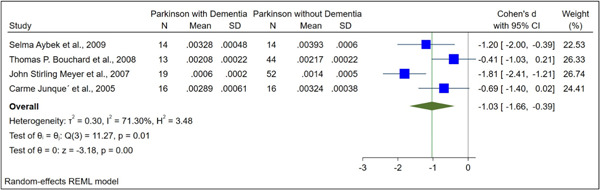
The forest plot of comparison of normalized hippocampal volume between PDD and PD, demonstrating statistical differences in the hippocampal volume. PD, Parkinson's disease; PDD, Parkinson's disease with dementia.

The basic characteristics of the included studies are illustrated in Table 1. The normalized and raw baseline volumes of the hippocampal region were assessed in nine studies among patients with PDD and PD. In terms of the raw hippocampal volumes, there was an overall SMD between PDD and PD of −0.53 (−1.02 to −0.05) (Figure 2). Regarding the normalized hippocampal volumes, the total SMD between PDD and PD was −1.03 (−1.66 to −0.39) (Figure 3). PD patients had higher hippocampal volumes than those with PDD, except in one study.^12^

**Table 1:** The systematic review of included studies.

Studies' info				PD patients with dementia			PD patients without dementia					
Code	Author	Country/year	Study design	Sample size (total/female/male)	Age	Hoehn and Yahr Scale Score	Sample size of (total/female/male)	Age	Hoehn and Yahr Scale Score	Studied regions (ROIs)	Findings	Acknowledgments
1	Gazzina et al.^11^	Italy/2016	Cohort	11/2/9	71.6 (5.6)	NR	16/3/13	69.4 (4.7)	NR	Hippocampal volume, caudate nucleus (normalized)	It might be helpful to study subcortical structures with structural MRI to define PD patients at risk for dementia and differentiate between PDD and DLB in cases where the diagnosis is uncertain.	There is a significant limitation in the statistical power of this study due to the small number of subjects and the need to covariate for parameters known to be influential on brain volume (e.g., age, gender, disease duration).
2	Kandiah et al.^7^	Singapore/2014	Prospective	8/NA/NA	NR	NR	36/NA/NA	NR	NR	Hippocampal volume (raw and normalized)	Hippocampal volume plays a significant role in developing MCI and dementia in PDD.	Although minimal, repeated administration of the cognitive battery at 6‐month intervals may not completely eliminate the possibility of learning effects. A unique factor in the population might be the local prevalence of vascular risk factors, as well as the local genetic composition. Detailed information on the treatment and control of vascular risk factors was lacking.
3	Lee et al.^12^	Korea/2013	Cross‐sectional	16/14/2	69.9 (6.5)	2.6 (0.6)	16/13/3	63.8 ± 7.2	1.7 ± 0.6	Hippocampal volume (raw)	In PD, hippocampus loss may not contribute to cognitive impairment. Structural changes in PDD may be related to variations in the regional volume within the frontal and parietal lobes.	Even though MMSE is a general screener for cognitive impairment, its sensitivity for diagnosing PDD is low.
4	Apostolova et al.^13^	Norway/2010	Cohort	15/5/10	73.4 (7.6)	3.2 (0.7)	12/6/6	68.4 (6.8)	2.3 (0.4)	Hippocampal volume, right and left lateral ventricles, caudate nucleus (raw)	There is an association between cognitive impairment in Parkinson's disease, anterior caudate atrophy, and an enlargement of the ventricles.	There is a need for further studies to examine possible relationships between visuospatial performance and ventricular enlargement, memory and hippocampal atrophy, or executive dysfunction and anterior caudate atrophy.
5	Aybek et al.^14^	Switzerland/2009	Cohort	14/5/9	69.2 (5.7)	2.46 (0.5)	14/5/9	66.6 (6.6)	2.46 (0.6)	Hippocampal volume (raw and normalized)	In the case of Parkinson's disease, hippocampal atrophy could be a predictor of the development of dementia.	There is still a need for more extensive studies to be conducted to identify a cut‐off score for hippocampal atrophy that would allow accurate prediction of the evolution of the disease among individuals.
6	Bouchard et al.^15^	Canada/2008	Cross‐sectional	13/4/9	71.9 (5.9)	2.3 (0.7)	44/18/26	71.1 (4.3)	2.2 (0.6)	Hippocampal volume (normalized)	In Parkinson's disease, the hippocampal and amygdala have functionally significant age‐associated atrophy.	Future studies should examine specific extra‐hippocampal brain regions, which this study has not examined.
7	Meyer et al.^16^	USA/2007	Longitudinal	19/11/8	78.74 (5.05)	NR	52/28/24	65.6 (11)	NR	Hippocampal volume (normalized), third ventricle volume (normalized), frontal horn volume (normalized), temporal horn volume (normalized)	There are subtypes of MCIs that are precursors to different types of dementia. A distinctive structural change on MRI is also distinguishable between these different MCI subtypes.	This study's MCI and dementia subjects were significantly older than cognitively normal subjects. Thus, MRI differences in CN and other groups may be overestimated because normal aging also affects brain structures.
8	Meyer et al.^16^	USA/2007	Longitudinal	17/7/10	77.47 (6.82)	NR	52/28/28	65.6 (11)	NR	Hippocampal volume (normalized), third ventricle volume (normalized), frontal horn volume (normalized), temporal horn volume (normalized)	‐	‐
9	Meyer et al.^16^	USA/2007	Longitudinal	5/2/3	76.02 (3.03)	NR	52/28/28	65.6 (11)	NR	Hippocampal volume (normalized), third ventricle volume (normalized), frontal horn volume (normalized), temporal horn volume (normalized)	‐	‐
10	Junqué et al.^6^	Spain/2005	Cross‐sectional	16/11/5	70.06 (7.9)	3.37 (1.1)	16/10/6	72.87 (7.9)	2.69 (0.68)	Hippocampal volume (raw and normalized)	In PDD, the hippocampal and amygdalar volumes decrease. MRI can reveal limbic involvement in PD, and limbic atrophy is evident in both demented and nondemented patients	‐
11	Laakso et al.^17^	Finland/1996	Cross‐sectional	8/5/3	71 (2)	NA	12/6/6	68 (5)	NR	Hippocampal volume (raw)	In clinical practice, hippocampal atrophy may be a valuable marker for AD, but the specificity of this atrophy limits it.	‐

Abbreviations: AD, Alzheimer's disease; DLB, dementia with Lewy body; MCI, mild cognitive impairment; MMSE, mini‐mental status examination; NR, not reported; PD, Parkinson's disease; PDD: Parkinson's disease with dementia.

### 3.3 Comparison of raw hippocampal volume between PDD and PD

Five studies compared the raw hippocampal volumes between PDD (*N* = 69) and PD (*N* = 70) patients. The total number of female and male participants was 80 and 59, respectively. The mean age of PDD and PD participants was 70.68 ± 6.6 and 68.97 ± 7.02, respectively. A statistically significant difference was observed comparing the raw hippocampal volumes in participants with PDD and PD (SMD: −0.53, 95% confidence interval, CI [−1.02 to −0.05], *p* value = 0.01; Figure 2).

### 3.4 Comparison of normalized hippocampal volume between PDD and PD

Four studies compared PDD (*N* = 62) and PD (*N* = 96) based on normalized hippocampal volume. There were 92 female and 157 male patients in total. Participants with PDD and PD had a mean age of 72.91 ± 7.26 and 68.55 ± 8.74, respectively. In these studies, ROI was divided by ICV to normalize volumes. In a comparison of normalized hippocampal volume between PDD and PD, there was a statistically significant difference (SMD: −1.03, 95% CI [−1.66 to −0.39], *p* value < 0.001; Figure 3).

### 3.5 Publication bias

**Figure 4:**
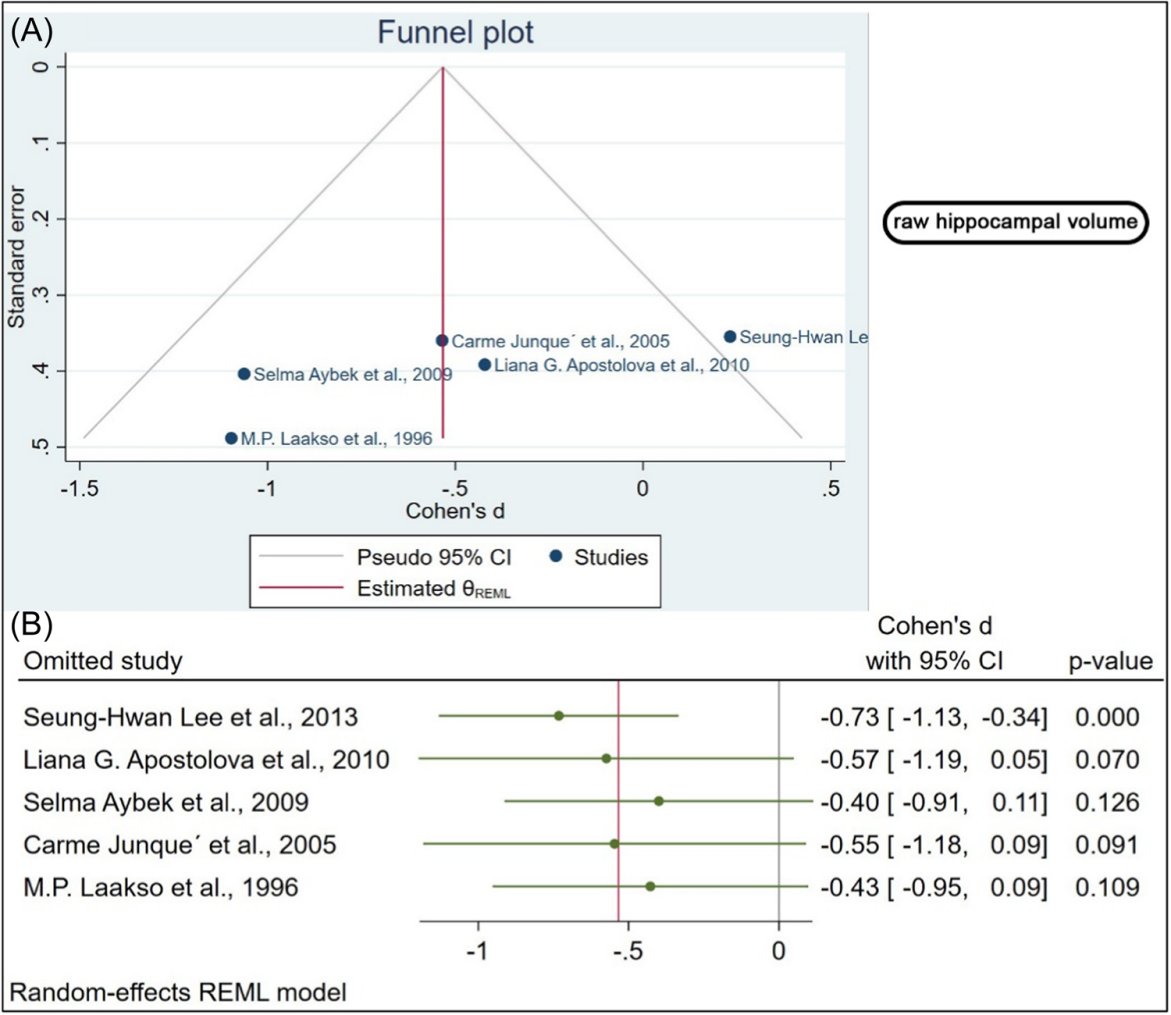
(A) The funnel plot provides no evidence of publication bias, and Egger's regression test confirms this. (B) Sensitivity analysis indicates that omitting four studies significantly alters the effect size.

**Figure 5:**
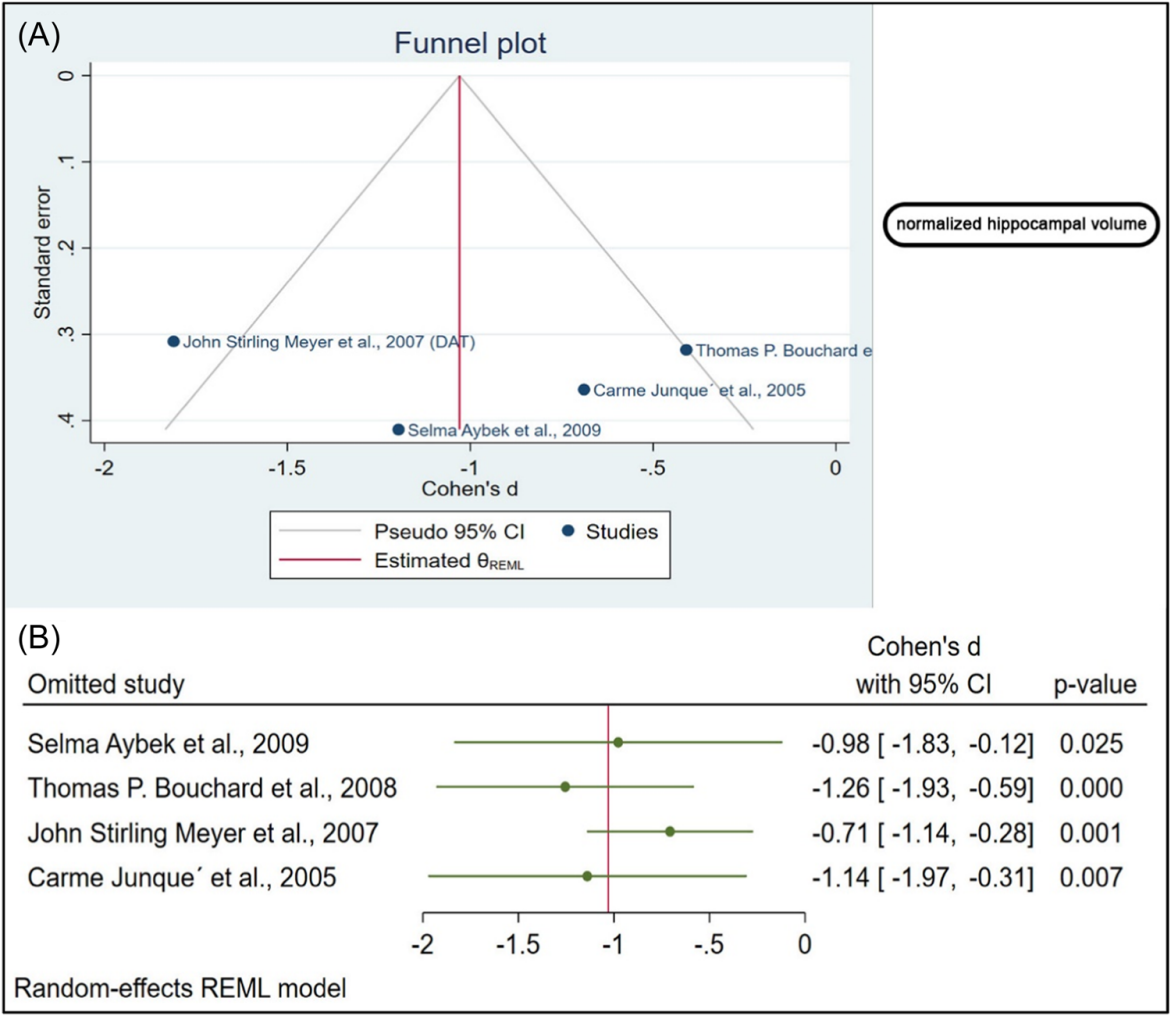
(A) The funnel plot demonstrates no publication bias, as confirmed by Egger's regression test. (B). Sensitivity analysis found no significant changes.

Using the funnel plot, we showed no evidence of publication bias, which was statistically confirmed by Egger's regression test, done separately for the normalized (*β*1 = 1.39, *p* value = 0.1) and the raw hippocampal volumes (*β*1 = − 8.21, *p* value = 0.1; Figures 4A and 5A).

### 3.6 Sensitivity analysis

We conducted a sensitivity analysis to determine whether the influential cases impacted the significance level of the meta‐analysis. The raw hippocampal volume was analyzed as part of the sensitivity analysis. A significant change in the *p* value was observed after removing four studies.^7^, ^9^, ^10^, ^12^ In the absence of one study, the biggest difference in *p* value was observed (Figure 4B).^10^ Regarding Normalized hippocampal volume, the effect size was calculated each time by omitting one study from the analysis and recalculating it. When the studies were removed one by one, no significant changes were found in the sensitivity analysis (Figure 5B). According to this analysis, the smallest and highest effect size was (SMD: −1.04, 95% CI [−1.52 to −0.56], *p* value < 0.001) and (SMD: −0.71, 95% CI [−1.14 to −0.28], *p* value = 0.001) respectively (Figure 5B).

### 3.7 Univariate meta‐regression

To determine the cause of heterogeneity, a meta‐regression analysis was performed. It is important to note that, regarding the source of heterogeneity in the normalized hippocampal volumes, the total number of females and males did not contribute to the source of heterogeneity (*p* > 0.05); however, in the raw hippocampal volumes, the total number of females accounted for 100% of the heterogeneity (*p* < 0.05), whereas the total number of males did not account for the source of heterogeneity in the non‐normalized hippocampal volumes (*p* > 0.05).

## 4 DISCUSSION

The in vivo neuropathological basis of cognitive deficits associated with PD may be studied through MRI volumetric studies, even though they are currently limited in their clinical utility for dementia diagnosis. Also, MRI and postmortem studies have been used to report on hippocampal volumetric atrophy in PD.^6^, ^8^, ^17^ Our results showed that PDD patients showed significant reductions in the hippocampus, which is in line with previous postmortem volumetric studies. This meta‐analysis revealed a substantial decrease in hippocampus volume in PDD patients. A statistically significant difference was observed comparing the raw hippocampal volumes in participants with PDD and PD (SMD: −0.53, 95% CI [−1.02 to −0.05], *p* value = 0.01, Figure 2). In a comparison of normalized hippocampal volume between PDD and PD, there was a statistically significant difference (SMD: −1.03, 95% CI [−1.66 to −0.39], *p* value < 0.001, Figure 3).

The significant differences with more hippocampal volume reduction in PDD patients stress the effect of aging on the pathophysiology of PDD. Also, other age‐related pathologies, such as cerebrovascular pathology, amyloid pathology, and tau pathology, can be contributed to developing dementia in PDD patients.^7^

Histopathological investigations have found Lewy bodies and Alzheimer's‐like changes in the hippocampus in PDD patients, both plausible causes of cognitive impairment.^18^, ^19^ The CA2 and CA3 fields of the hippocampus are where Lewy bodies, Lewy neurites, and fibrillar tangles are most frequently observed.^20^, ^21^ In the CA1 region of the hippocampus, more neurofibrillary tangles have been observed in PDD patients.^19^ The influence of hippocampus degradation on the progression of dementia in PD was emphasized by Churchyard and Lees.^20^ The degenerative process associated with Lewy neurite development in CA2 impairs hippocampal function and cognition by interfering with inputs to the CA1 field. The number of Lewy neurites in the CA2 area of the hippocampus may be correlated with the severity of dementia.

In a study using voxel‐based morphometry (VBM) of demented patients,^22^ hippocampal atrophy was observed in PDD compared to PD, but the results for the amygdala were not statistically significant. These differences were also reported by Good et al.^23^ in a research that included Alzheimer's patients. In their investigation, ROI analyses appeared more sensitive to amygdala volume loss, whereas VBM seemed more sensitive to local hippocampal volume loss.

Our meta‐analysis possesses both strengths and limitations. The strength of our study is that we included all recent case‐control studies performed on the volume changes of the hippocampus in PD patients with and without dementia. A sensitivity analysis was also performed that identified the studies, which resulted in insignificant overall findings. The first limitation of our study is that in most studies that we included, the volume changes were noted in the brain's hippocampus. However, other parts, like the amygdala, were not considered. The small number of studies unable to do stratified analysis based on age‐ and sex‐matched control would be another limitation of our study.

## 5 CONCLUSION

After accounting for overall brain atrophy, PDD patients still exhibit statistically substantial hippocampal atrophy. Prospective research will examine whether this is a presymptomatic sign of dementia in PDD and whether the progression of atrophy in PD is similar to that reported in other neurodegenerative diseases. The pattern of brain atrophy, particularly in the medial temporal lobes, will be further, more precisely defined by ongoing studies employing better‐resolution MRI scans.

## AUTHOR CONTRIBUTIONS

**Mohammad Yazdan Panah**: Conceptualization; writing—original draft; writing—review and editing. **Yousef Mokary**: Conceptualization; supervision; visualization; writing—review and editing. **Sangam Shah**: Conceptualization; data curation; resources; visualization; writing—original draft. **Sangharsha Thapa**: Conceptualization; data curation; methodology; supervision; visualization; writing—review and editing. **Swati Chand**: Conceptualization; formal analysis; supervision. **Vahid Shaygannejad**: Conceptualization; investigation; supervision. **Omid Mirmosayyeb**: Conceptualization; investigation; methodology; supervision.

## CONFLICT OF INTEREST STATEMENT

The authors declare no conflict of interest.

## TRANSPARENCY STATEMENT

The lead author Sangharsha Thapa affirms that this manuscript is an honest, accurate, and transparent account of the study being reported; that no important aspects of the study have been omitted; and that any discrepancies from the study as planned (and, if relevant, registered) have been explained.

## Data Availability

All the required information is in the manuscript itself.
